# Maximizing Outcomes While Minimizing Morbidity: An Illustrated Case Review of Elbow Soft Tissue Reconstruction

**DOI:** 10.1155/2016/2841816

**Published:** 2016-05-29

**Authors:** Adrian Ooi, Jonathan Ng, Christopher Chui, Terence Goh, Bien Keem Tan

**Affiliations:** Department of Plastic, Reconstructive and Aesthetic Surgery, Singapore General Hospital, Singapore 169608

## Abstract

*Background*. Injuries to the elbow have led to consequences varying from significant limitation in function to loss of the entire upper limb. Soft tissue reconstruction with durable and pliable coverage balanced with the ability to mobilize the joint early to optimize rehabilitation outcomes is paramount.* Methods*. Methods of flap reconstruction have evolved from local and pedicled flaps to perforator-based flaps and free tissue transfer. Here we performed a review of 20 patients who have undergone flap reconstruction of the elbow at our institution.* Discussion*. 20 consecutive patients were identified and included in this study. Flap types include local (*n* = 5), regional pedicled (*n* = 7), and free (*n* = 8) flaps. The average size of defect was 138 cm^2^ (range 36–420 cm^2^). There were no flap failures in our series, and, at follow-up, the average range of movement of elbow flexion was 100°.* Results*. While the pedicled latissimus dorsi flap is the workhorse for elbow soft tissue coverage, advancements in microvascular knowledge and surgery have brought about great benefit, with the use of perforator flaps and free tissue transfer for wound coverage.* Conclusion*. We present here our case series on elbow reconstruction and an abbreviated algorithm on flap choice, highlighting our decision making process in the selection of safe flap choice for soft tissue elbow reconstruction.

## 1. Introduction

Soft tissue defects of the elbow are commonly encountered by the reconstructive surgeon and can result from causes such as trauma, infection, burns, tumour resection, and radiation injuries. The extent of injury can involve a wide variety of tissues including skin, vessels, nerves, muscle, bone, and joints. Reconstruction of these defects is a challenging conundrum which requires pliable and durable skin that can allow for repetitive flexion and extension. In addition, the elbow joint is prone to develop postinjury stiffness and early mobilization is of paramount importance for optimal recovery [1]. The final outcome in patients with complex elbow injuries is largely dependent on the extent of the initial injury, with larger and more complex injuries involving the bones and joints associated with a higher degree of stiffness [2]. Historically, soft tissue coverage around the elbow has used local and regional pedicled flaps. With advances in microsurgery, free tissue transfer is now the gold standard for composite defects and is often the most favorable choice for early return of function [3]. The option chosen for defect coverage is traditionally based on the exposure of critical structures and the size and location of the defect [4]. Poor decision making can result in reconstructive failure, which may lead to catastrophic outcomes such as osteomyelitis or amputation. Therefore a third dimension that must be considered is the safest path to success. At our institution, we have utilized a variety of flaps to achieve successful coverage while minimizing patient and wound morbidity. Using our clinical cases, this paper aims to review the reconstructive options available and highlight our decision making process which has helped to maximize clinical outcomes for the patient.

## 2. Methods

20 consecutive patients at our institution underwent soft tissue flap coverage of the elbow for coverage of critical structures. Wounds were classified according to size and location. Flaps types used for reconstruction were divided into three categories: local flaps, regional pedicled flaps, and free flaps. Local flaps encompass all perforator-based fasciocutaneous flaps (including cases where tissue expansion was utilized) as well as local muscle flaps. Indications included trauma (*n* = 10), infection (*n* = 4), tumour resection (*n* = 3), exposed orthopedic implants (*n* = 2), and myositis ossificans (*n* = 1). The average defect size was 138 cm^2^ (range 36–420 cm^2^). The mean age of the patients was 40 years (range 25–61). 16 patients were male and 2 were female. Average time from injury to coverage was 18.8 days (range 11–42).

## 3. Results

**Figure 1:**
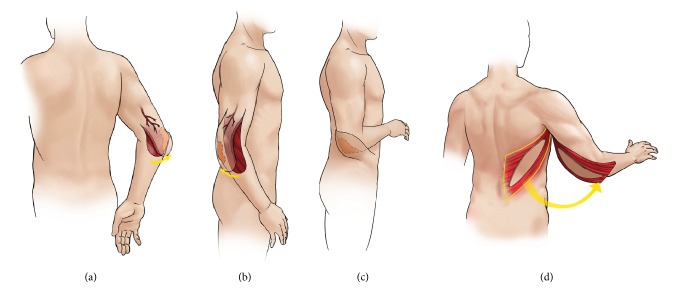
(a) Perforator-based medial fasciocutaneous transposition flap. (b) Perforator-based lateral forearm flap. (c) Pedicled lateral abdominal perforator flap. (d) Pedicled latissimus dorsi flap.

Patient data is summarized in Table 1. Flap types were divided into local (*n* = 5), pedicled (*n* = 7), and free flaps (*n* = 8). Average defect size for the local flaps was 59.4 cm^2^ (range 40–75 cm^2^). Perforator-based local flaps used included 1 medial rotational and 1 pedicled lateral forearm. We used 1 pedicled flexor carpi radialis (FCR) and flexor carpi ulnaris (FCU) muscle flap. There were 2 cases of tissue expansion and advancement. For the regional pedicled flaps, average defect size was 119 cm^2^ (range 80–150 cm^2^). This category of flaps included 2 latissimus dorsi (LD) (Figure 1(d)) flaps, 2 extended groin flaps, 1 lateral abdominal perforator flap, and 1 rectus abdominis muscle flap. Average defect size for the free flaps was 213 cm^2^ (range 36–420 cm^2^). We used 7 anterolateral thigh (ALT) flaps, 1 free LD myocutaneous flap, and 1 free rectus abdominis flap.

Postoperative complications included 3 cases of wound breakdown, one in a free ALT flap and one in a pedicled LD muscle flap and one superficial infection in the lateral forearm flap. There was one major complication of septic arthritis in a free ALT flap patient, which necessitated the removal of orthopedic implants. There were no flap failures. At an average follow-up of 12 months, the range of movement for 19 patients recorded on a goniometer was an average of 100° (range 45–140°). One patient was lost to follow-up.

### 3.1. Case Examples

**Figure 2:**
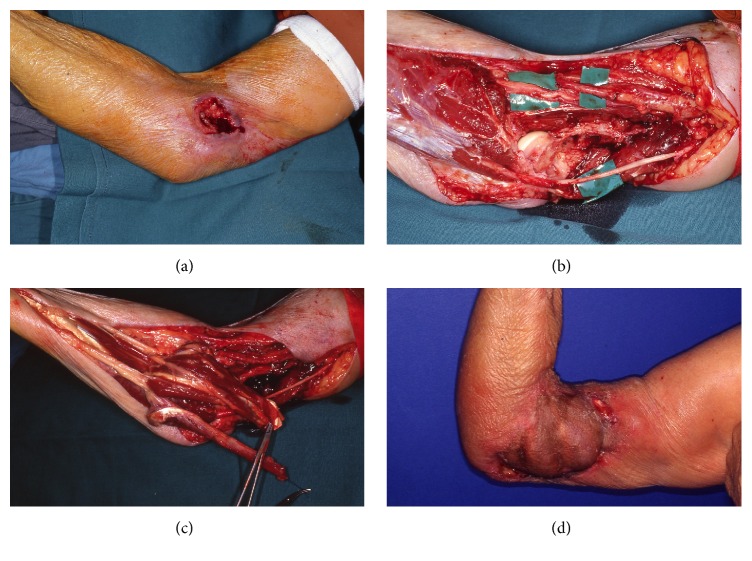
(a) A squamous cell carcinoma measuring 5 × 4 cm over the medial elbow of a 75-year-old female. (b) Defect after resection: the brachial artery, median nerve, ulnar nerve, and elbow joint are exposed. The humeral epicondyle together with the origins of the flexor carpi ulnaris, flexor carpi radialis, and pronator teres was resected. (c) The flexor carpi ulnaris and flexor carpi radialis muscles were transposed to cover the defect and skin grafted. (d) Postoperative result after 4 months.

*Case  1*. An 80-year-old female with multiple comorbidities including ischemic heart disease and chronic renal failure developed an ulcerating squamous cell carcinoma of her right elbow measuring 7 × 5 cm. Radiological studies showed no bony or joint involvement. The resultant wide excision included the skin, subcutaneous tissue, and underlying muscle, with a defect size of 75 cm^2^ exposing the elbow joint capsule and ulnar nerve. To avoid prolonged operative time, the defect was covered with pedicled FCR and FCU muscles (Figure 2). The postoperative course was uneventful and in 10 months postoperatively the range of motion (ROM) at the elbow was 90°.

**Figure 3:**
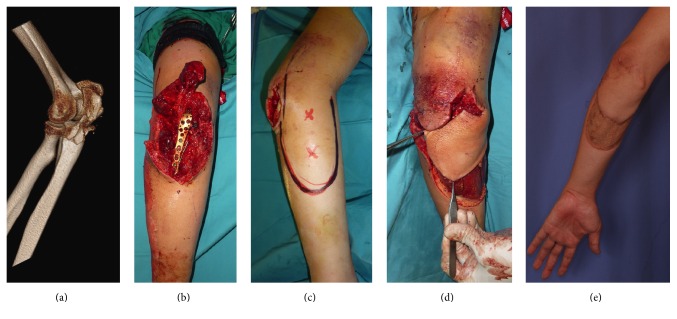
Perforator-based transposition flap. ((a), (b)) A 29-year-old man sustained an open olecranon fracture for which plate fixation was done. ((c), (d)) A proximally based medial forearm fasciocutaneous transposition flap was used to cover the elbow defect. (e) Postoperative result in 1 year. The donor site defect was skin grafted.

*Case  2*. A 29-year-old male sustained an open olecranon fracture of the left elbow. Fixation of the fracture was done resulting in a 60 cm^2^ soft tissue defect. This was covered with a medial fasciocutaneous transposition flap based on a medial upper arm perforator. The donor site was skin grafted. Recovery was uneventful, and the patient's ROM at the elbow was recorded as 100° 8 months postoperatively (Figure 3). 

**Figure 4:**
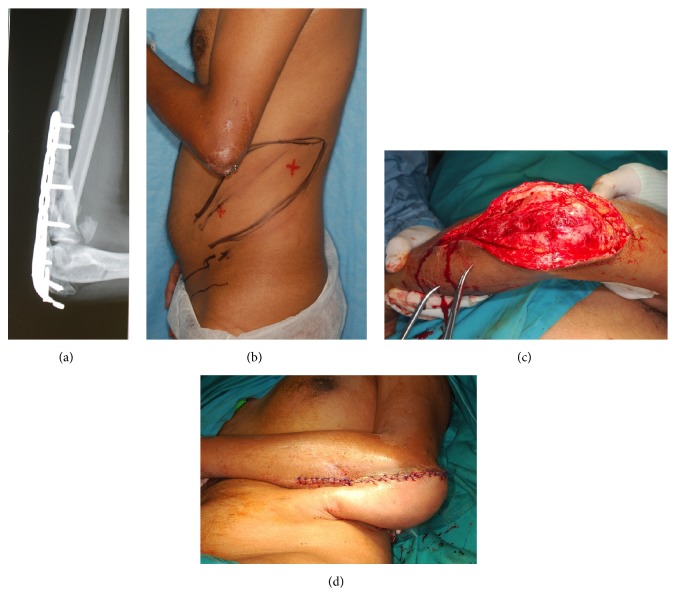
Pedicled lateral abdominal perforator flap. ((a), (b)) 30-year-old male with fixed flexion deformity of the elbow and exposed plate. He had traumatic brachial plexus and brachial artery injuries. (c) Defect after removal of the implant. (d) A pedicled lateral abdominal perforator flap was used to cover the defect.

**Figure 5:**
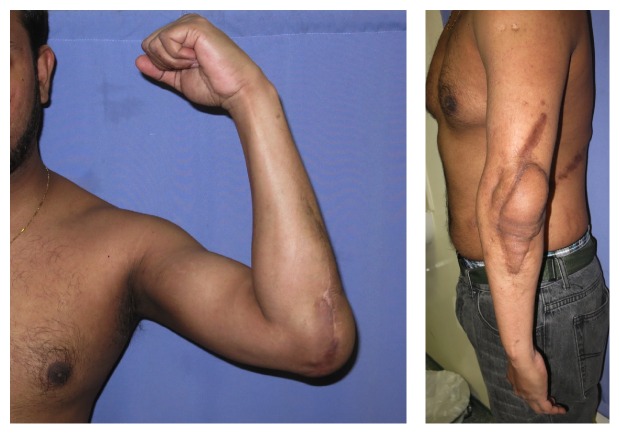
Same patient as in Figure 4 in 10 months postoperatively with 70° elbow range of movement, similar to preop.

*Case  3*. A 30-year-old male sustained a left elbow fracture with severed brachial artery for which vascular repair of the artery and fixation of the fracture were done. Nine months later he presented with exposure of the implant. He was very thin and had significant heterotopic calcification causing ankylosis of the elbow after injury. Due to the ankylosis and previous brachial artery injury exploration of vessels for free tissue transfer was difficult; therefore lateral abdominal perforator flap was used to cover the defect after implant removal (Figures 4 and 5). The coverage required 2 stages and was completed within 3 weeks. Ten months postoperatively his ROM was 70°, which was similar to his preoperative ROM.

**Figure 6:**
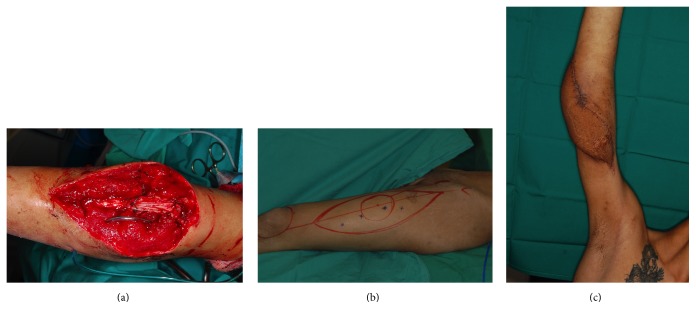
(a) Open fracture of the posterior elbow in a 26-year-old male. (b) A left free anterolateral thigh flap was used to resurface the soft tissue defect. (c) Postop result in 8 months.

*Case  4.* A 35-year-old male sustained loss of anterior elbow soft tissue in a workplace accident. This exposed underlying tendons, vessels, and nerves. The large defect was resurfaced with a free ALT fasciocutaneous flap with a cuff of vastus lateralis muscle (Figure 6). In 12 months postoperatively, the patient has excellent contour and 110° ROM.

## 4. Discussion

**Figure 7:**
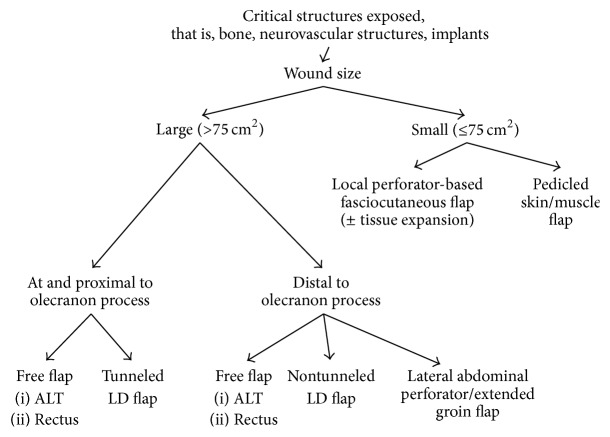


Reconstruction of the elbow is a challenging subject. In our series, we were able to achieve a balance of successful pliable soft tissue coverage and early mobilization of the joint which led to good postoperative function. All this was achieved with no flap failures and a small number of complications whose occurrences were independent from flap choice. Flap coverage in our center depended on a number of variables, including size of the wound, patient comorbidities, surgical expertise, and vital structures involved. We successfully applied our abbreviated algorithm presented in Figure 7 to achieve a series of safe and efficacious flap choice in our 20 patients requiring elbow reconstruction.

Local fasciocutaneous flaps are simple and fast, suitable for small shallow defects with healthy adjacent tissue. These flaps enabled the earliest mobilization of the elbow joint in our series of patients and allowed the patients a relatively quicker return to full range of motion. In cases where timing of coverage is not an issue and additional tissue is required, tissue expansion can be carried out as a staged surgery as was done in two of our patients. While local fasciocutaneous flaps were originally of random pattern in nature, the angiosome concept first described by Taylor et al. forms the basis for a predictable axial pattern of blood supply to the skin and fascia via fasciocutaneous or musculocutaneous perforators [5]. Local axial fasciocutaneous flaps have the advantage of a known axial blood supply, which allows for more mobility and a narrower flap base. Axial flaps to the elbow are based on the radial, ulnar, anterior, and posterior interosseous arteries with a rich interconnecting vascular network [5–7]. These vessels give off perforators to the skin and forearm at regular intervals and form the basis for retrograde flaps to the elbow. The most common axial fasciocutaneous flap for elbow coverage in the literature is the radial forearm flap [8]. The radial forearm flap possesses a flexible arc of rotation, reliable vascularity, and possible sensory innervation. In our series we used a perforator-based medial transposition flap (Figures 1(a) and 3) and a perforator-based lateral forearm flap (Figures 1(b) and 4). The drawbacks of these flaps are their limited mobility and size and donor site scarring.

There are numerous local muscle flaps available for coverage, such as the flexor carpi ulnaris, brachioradialis, and anconeus flaps [9–11]. Some flaps, such as the brachioradialis, have been described with a skin island. Each has its benefits and drawbacks, but apart from the anconeus muscle flap ultimately each of these options does sacrifice some level of function. Key to utilizing local muscle is to balance need for local coverage with absence of fasciocutaneous tissue with donor site morbidity. It is not our preference to use local muscle as we feel the donor site morbidity warrants the use of regional pedicled flaps instead. We used pedicled FCU and FCR muscles in a single case where a patient with multiple comorbidities and lack of local fasciocutaneous tissue for coverage required a simple and expedient solution to coverage (Figure 1). The literature has shown that local tissue can be used to cover defects averaging ranging from 12 cm^2^ to 164 cm^2^, with an average of 55 cm^2^. The majority of these flaps covered defects less than 80 cm^2^, with the exception of the ulnar recurrent adipofascial flap, which has been shown to cover defects up to 164 cm^2^. Stevanovic and Sharpe have recently presented their case based series of elbow reconstructive options with a preference towards local-regional flap options due to their experience with donor site morbidity, such as chronic aching, dysesthesias, and poor cosmetic appearances [12].

Historically, distant fasciocutaneous pedicled flaps were commonly used for elbow coverage [13]. These were usually raised from the groin or chest wall and required multiple operations including division and inset. These flaps have largely been abandoned because of multiple disadvantages including long hospital stay and joint stiffness due to prolonged immobilization. However, we feel that they are still a viable alternative for patients with medium-sized defects and in those who do not have other options of free tissue transfer or local flaps. These patients either have no recipient vessels or previous vessel injury, brachial plexus injuries or have minimally functioning elbow joints with preexisting Volkmann's contracture, thus minimizing the necessity for early mobilization. These regional fasciocutaneous flaps can be made even more reliable with a delay procedure. Examples of flaps that we have used in the past include the extended groin flap based on the superficial circumflex iliac artery (Figures 4 and 5) and the lateral abdominal perforator flap (Figure 1(c)) based on the lateral abdominal wall perforators. The latter was done in a patient where severe elbow joint injury had caused brachial artery injury, making free tissue transfer difficult. In addition to this, he had posthealing heterotopic calcification at the elbow joint which led to severe ankylosis and permanent deformity. On balance, a distant pedicled fasciocutaneous flap provided the simplest method of coverage in a patient with poor elbow rehabilitative function.

Muscle flaps for soft tissue coverage are preferred when infection is present or when muscle bulk is required for obliteration of significant dead space [14]. In addition to providing coverage, flaps such as the pedicled latissimus dorsi or triceps may be transferred to restore elbow flexion. In our experience, we have used tunneled and nontunneled pedicled latissimus dorsi flaps as well as a single case of a pedicled proximally based rectus abdominis flap. The latissimus dorsi muscle flap is the workhorse flap for elbow soft tissue coverage and is preferred because of its size, versatility, ease of use, and reliability [15–18]. It can be mobilized to include either part of the muscle or the entire muscle and can include a skin paddle for cutaneous resurfacing. In addition, the donor site can be closed primarily. However, when used for defects beyond the olecranon it has a propensity for distal tip necrosis, wound breakdown, or failure [18]. The resultant wound edge tension from extension beyond the olecranon may also require extended periods of immobilization.

Microsurgical techniques have revolutionized the field of reconstructive surgery by allowing reliable transfer of free soft tissue to replace muscle, skin, or bone in small or large defects. Although free tissue transfer has become increasingly popular, the literature contains relatively few reports on its use in elbow coverage. Hallock advocated using local flaps for mild injuries around the elbow but maintained that free flaps were necessary in larger or composite defects [19]. Choudry et al. reported using free tissue transfers in only 19 percent of cases in their series of 96 patients requiring soft tissue coverage of the elbow [2].

The advantages of free flaps are manifold. They are versatile and can afford coverage to large wounds. They are especially useful when trauma to surrounding tissues has excluded the use of local flaps or when external fixation precludes local flap use. The flap can be harvested from a multitude of locations in various forms. Other than the ALT flap, fasciocutaneous flaps including the thoracodorsal artery perforator, superficial circumflex iliac artery perforator, scapular, parascapular, and lateral arm flaps offer good skin coverage [20–25]. Some authors advocate the use of muscle flaps when infection is present or significant dead space needs to be filled [13]. Potential donor sites for pure muscle or myocutaneous flaps include the latissimus dorsi, rectus abdominis, or gracilis. Free flaps are also useful when composite tissue needs to be replaced, such as when tendon reconstruction or vascularized bone is required, for example, in a vascularized tensor fascia lata flap. This allows restoration of both form and function simultaneously. Free tissue transfer also tends to produce donor sites that can be closed primarily. Another advantage of free flaps is the potential for functional muscle transfer to restore elbow flexion. The use of free tissue does, however, relies on the availability of recipient vessels. In our experience, revascularization of the flap is best done end-to-end to the radial or ulnar artery by turning their proximal ends up or end-to-side to the brachial artery if vessels distant to the joint are not available.

Choice of flap is dictated by size of defect, donor site morbidity, and tissue defect. In the past, we have used a rectus abdominis flap to cover a large elbow defect. More recently, Chui et al. reported the use of the anterolateral thigh (ALT) flap in 5 patients from our center [24]. Defects ranging from 36 cm^2^ to 450 cm^2^ were resurfaced with either fasciocutaneous or musculocutaneous ALT flaps, with no flap failures or major complications. All patients had reasonable and functional return of active elbow motion. The advantages of the ALT flap are large amounts available and the potential to include the vastus lateralis in the flap. The motor nerve to the vastus lateralis can be used as a vascularised nerve graft if required, and fascia lata grafts can be harvested at the time of flap elevation. In addition, the fasciocutaneous flap allows for gliding of tendons and secondary surgery if needed.

We recognize that the above study is limited by its small sample size. However, each case was carefully considered and dissected based on each individual's requirements. In addition to this, our case series and algorithm have been limited to the need for soft tissue reconstruction of the elbow. Patients who require bone or joint reconstruction would need additional considerations besides those mentioned above.

In our center, the presence of microsurgical expertise in combination with the versatility of free tissue transfer has now made it our first choice for soft tissue coverage in the reconstruction of large elbow defects. Drawing from our clinical experience and literature review, we have created a clinical algorithm (Figure 7) for soft tissue coverage of the elbow that is based on size and location of the defect which has helped our center make safe yet rehabilitation-optimized flap choices. When critical structures are exposed, soft tissue coverage is paramount and the choice of flap is simply mandated by size. Smaller defects, determined from our experience as those <75 cm^2^, can be covered with local skin and/or muscle whilst defects ≥75 cm^2^ warrant either pedicled regional or free flap coverage. Though, all of the above reconstructive options may be used for soft tissue reconstruction of the elbow. We have found, when considering the factors presented above, we have been able to provide appropriately sized, durable coverage for our patients, with the additional benefit of availing them to rehabilitative specialists at the earliest possible time, whilst minimizing the risk of complications.

## Figures and Tables

**Table 1:** Summary of patient data.

Age	Sex	Flap type	Cause	Size (cm^2^)	Complications	Range of motion (degrees)
Local flaps						
29	M	Medially based transposition	Trauma	60	Nil	100
37	M	Advancement	Myositis ossificans	50	Nil	120
40	F	Advancement	Prominent plate	40	Nil	130
38	M	Lateral forearm	Trauma	72	Superficial infection	100
50	F	Flexor carpi radialis muscle, flexor carpi ulnaris muscle	Tumour	75	Nil	90

Regional pedicled flap						
39	M	Extended groin	Trauma	104	Nil	90
30	M	Extended groin	Trauma	117	Nil	Lost to follow-up
30	M	Lateral abdominal perforator	Exposed implant	150	Nil	70
58	M	LD myocutaneous	Postop infection	80	Wound breakdown	85
40	F	LD muscle	Trauma	120	Nil	110
45	M	LD muscle	Tumour	140	Nil	100
50	M	Rectus	Trauma	140	Nil	105

Free flaps						
40	M	ALT	Trauma	420	Nil	115
35	M	ALT	Trauma	360	Nil	110
40	M	ALT	Trauma	240	Septic arthritis	45
25	M	ALT	Postop infection	120	Wound breakdown	100
61	M	ALT	Postop infection	36	Nil	140
56	M	Rectus	Postop infection	104	Nil	80
